# Editorial: Progressing the understanding and management of bloodstream infections

**DOI:** 10.3389/fmed.2025.1562934

**Published:** 2025-02-04

**Authors:** Lihui Wang, Bin Lin, Xingrong Gong, Yuetian Yu

**Affiliations:** ^1^Department of Critical Care Medicine, Renji Hospital, School of Medicine, Shanghai Jiao Tong University, Shanghai, China; ^2^Key Laboratory of Intelligent Pharmacy and Individualized Therapy of Huzhou, Huzhou, China; ^3^Department of Pharmacy, Changxing People's Hospital, Second Affiliated Hospital of Zhejiang University School of Medicine, Huzhou, China; ^4^Department of Medical Administration, Renji Hospital, School of Medicine, Shanghai Jiao Tong University, Shanghai, China

**Keywords:** bloodstream infections, intensive care unit, antibiotics, antimicrobial stewardship programs, precision medicine

Bloodstream infections (BSIs) stand as a significant contributor to the global burden of infectious diseases, impacting both health and mortality on a widespread scale. Beyond the pivotal role of positive blood culture in confirming BSIs presence, healthcare providers grapple with myriad contributing factors. Consequently, an imminent imperative exists to explore novel avenues for diagnosing and treating BSIs, encompassing initiatives such as antimicrobial stewardship programs.

Therefore, we have compiled this Research Topic on “*Progressing the Understanding and Management of Bloodstream Infections*”, aiming for an in-depth discussion in the relevant fields. We are also pleased to receive a lot of submissions, and some of them have been successfully published after revisions. This Research Topic mainly focuses on the following areas.

## Application of blood culture and rapid diagnostic tests

Although molecular diagnostic technology has developed rapidly over the past decade, the gold standard for diagnosing BSIs remains blood culture. The traditional manual blood culture systems have evolved into automated systems since the late 1960s. With continuous technological improvements, sensitivity has further increased, and the turnaround time (TAT) has gradually shortened. Many medical institutions are now routinely equipped with blood culture enrichment instruments and automated inoculation systems, significantly enhancing the diagnostic efficiency and achieving 24-h fully automated management.

With the continuous advancement of molecular diagnostic technologies, an increasing number of rapid diagnostic tests (RDTs) have been applied clinically, such as dd-PCR, FilmArray, and GeneXpert. Most of these testing methods are based on the principle of nested PCR, allowing for rapid detection of a variety of pathogens. Clinicians can select one or more pathogen panels for quick testing based on the specific changes in the medical condition, typically obtaining results within 3 to 4 h. The differences in pathogens and drug-resistant phenotypes causing BSIs before and after the COVID-19 pandemic highlight the importance of these RDT technologies (1). They not only enable rapid identification of pathogens but also allow for the detection of drug-resistant phenotypes, contributing to the reduction of time from empirical treatment to targeted therapy. Currently, the Asia-Pacific region has developed expert consensus on the application of RDTs based on the Delphi principle, resulting in 10 suggestions to facilitate better clinical implementation of RDT, while ensuring quality control and reasonable interpretation of reports (2).

At the same time, for patients with a high suspicion of BSIs but negative culture results or suspected mixed infections with multiple pathogens, mNGS remains one of the best diagnostic technologies for identifying pathogens. It has been widely used in immunocompromised hosts, such as those with malignant hematological tumors and sepsis, and has improved the clinical prognosis of such patients (3–6). Other point-of-care testing (POCT) technologies based on rapid nucleic acid detection are also continuously being developed and applied, and relevant application consensus has been issued (7).

## Precise adjustment of antibiotic dosage

BSIs often affect the host's immune function, primarily due to the release of a large number of inflammatory mediators (such as cytokines and chemokines) within the patient's body, leading to a systemic inflammatory response. This excessive inflammatory response can damage normal tissues and organs, inhibiting the body's immune function. At the same time, BSIs can lead to increased apoptosis of immune cells (such as T cells and B cells), reducing the number of effective immune cells, thereby weakening the body's immune response capability. If the patient's adaptive immune response to the BSIs is decompensated, it may induce the occurrence of sepsis. Therefore, in addition to promptly initiating effective antibiotics, rapidly achieving the effective concentration of these antibiotics is one of the significant challenges in the treatment. In critically ill patients, especially those with BSIs, the pathophysiology can change rapidly. Factors such as apparent volume of distribution (Vd), serum albumin levels, changes in liver and kidney function, and extracorporeal life support devices can all impact the effective concentration of antibiotics (8). Thus, it is particularly important to guide antibiotic dosing based on therapeutic drug monitoring (TDM), as this not only optimizes the use of antimicrobial agents to reach ideal pharmacokinetic/pharmacodynamic (PK/PD) targets, but also improves clinical outcomes for patients with BSIs, reduces the occurrence of antibiotic resistance, and extends the clinical lifespan of currently available drugs. This has been a common pursuit of ICU physicians, clinical pharmacists, and clinical microbiology experts in recent years.

DALI studies have found that for patients with severe infections in the ICU, including BSIs, the empirical dosing of antibiotics may lead to 70% of patients not reaching appropriate therapeutic concentrations. Additionally, 50.4% of patients require dosage increases, while 23.7% need dosage reductions. Particularly for beta-lactam antibiotics, 16% of patients did not achieve the target of 50% f T>MIC, resulting in a 32% decrease in clinical response rates. The main reason is that patients with BSIs or sepsis initially require large volumes of fluid resuscitation, typically 30 ml/kg, which significantly increases the apparent Vd. Additionally, during the early stages of severe infection, there is a strong inflammatory response, and the body is in a state of high cardiac output (CO). This may also be accompanied by augmented renal clearance (ARC), which accelerates the clearance of antibiotics. Therefore, TDM becomes increasingly important.

Furthermore, more studies indicate that empirical dosing of antifungal agents also risks failing to reach target concentrations (9, 10). Consequently, the European Society of Intensive Care Medicine (ESICM) has led the publication of a position article regarding antimicrobial therapeutic drug monitoring in critically ill adult patients, recommending that TDM is strongly advised for various antibiotics including aminoglycosides, beta-lactams, glycopeptides, oxazolidinones, and voriconazole during anti-infection treatment. It is also suggested that TDM for Co-trimoxazole and polymyxins should be done if it is possible (11). As a result, many countries and regions have established a TDM alliance to share laboratory resources, aiming for timely and accurate monitoring of blood concentrations for various antibiotics. However, the application of TDM in patients with severe infections still faces many unresolved issues (Table 1), and further large-sample clinical studies are needed to provide additional evidence-based medical data.

**Table 1:** Issues that require further research in the field of therapeutic drug monitoring in the future.

1. How to provide timely and accurate TDM reports
2. How to develop TDM tools that can be implemented at the bedside
3. How to measure TDM targets for patients with bloodstream infections disseminated to other sites
4. How to determine the appropriate TDM target for antibiotic prophylaxis
5. How to determine the appropriate TDM target for the local use of antibiotics
6. How to develop TDM algorithms based on different individuals
7. How to better apply AI in the field of TDM
8. How to promote the widespread application of MIPD in clinical practice
9. How to share TDM resources across multiple medical centers
10. How to conduct multidisciplinary education and popularize TDM concepts

AI, Artificial Intelligence; MIPD, Modle-Informed Precision Dosing; TDM, Therapeutic Drug Monitoring.

## Rationalization of antimicrobial treatment duration

For critically ill patients with BSIs, it is essential to first eliminate the source of infection, such as removing contaminated intravascular devices and draining abdominal pus. Following the initiation of empirical antimicrobial therapy, the clinical response of the patient should be monitored while awaiting pathogen identification results, aiming for targeted treatment and early de-escalation of therapy. Early clinical studies have primarily focused on the timing of antibiotic initiation and the spectrum of antimicrobial activity, with few studies addressing the duration of antibiotic therapy for bloodstream infections. In the current era of multidrug-resistant organisms, it has become evident that prolonged use of antibiotics can induce resistance in pathogens, increase the burden on patients' liver and kidney functions, and disrupt the microbiome balance within the body. Therefore, short-course therapy is advocated. This involves using a short course of antibiotics in adult patients whose sources of infection have been adequately controlled, rather than a long course, including cases of bacteremia (12). Early studies have mainly concentrated on pneumonia and intra-abdominal infections, with minimal focus on BSIs. A recent multicenter, non-inferiority study included 3,608 hospitalized patients with BSIs (including those in ICU), who were randomly assigned to receive either 7 or 14 days of antimicrobial treatment. The results indicated that 7 days of antimicrobial therapy was not inferior to 14 days, thus providing further evidence for the safety of short-course therapy (13).

## Summary of the Research Topic and future directions

BSIs are the most severe type of infectious disease. During treatment, it is essential to strive for early identification of the pathogens causing the infection and their resistance profiles. At the same time, it is crucial to initiate empirical antibiotic therapy early, maximizing the achievement of PK/PD targets while formulating individualized treatment strategies and durations to improve patient prognosis. Currently, many new antibiotics are under development, some of which, such as ceftobiprole, have already been applied clinically for complicated BSIs caused by drug-resistant bacteria (14). Additionally, AI-based early warning models and Bayesian model-guided drug concentration monitoring have demonstrated significant clinical value in the management of the disease (15). In the future, the handling of severe complicated BSIs will continue to be guided by multidisciplinary team collaboration, enhancing cooperation among team members to ultimately improve patient outcomes.
